# Emerging infection: streptococcal toxic shock-like syndrome caused by group B Streptococcus (GBS), *Streptococcus agalactiae*

**DOI:** 10.4322/acr.2024.497

**Published:** 2024-06-21

**Authors:** Fareed Rajack, Shawn Medford, Ali Ramadan, Tammey Naab

**Affiliations:** 1 Howard University Hospital, Department of Pathology and Laboratory Medicine, Washington, D.C., United States of America; 2 Howard University College of Medicine, Washington, D.C., United States of America

**Keywords:** Amputation, Surgical, Bacteremia, Fasciitis, Necrotizing, Shock, Septic, Virulence Factors

## Abstract

*Streptococcus agalactiae* or Group B *Streptococcus* (GBS) infections are commonly associated with infections in neonates and pregnant women. However, there has been a rising incidence in nonpregnant adults. The risk of GBS infection in nonpregnant adults is increased for patients of advanced age and those with underlying medical conditions such as diabetes mellitus and cancer. We present a 77-year-old female with type-2 diabetes mellitus, hypertension, and bilateral foot ulcers that presented in probable septic shock with necrotic foot ulcers and necrotizing fasciitis and underwent bilateral lower limb amputations. The patient fulfilled the Streptococcal Toxic Shock Syndrome (STSS) criteria as defined by The Working Group on Severe Streptococcal Infections. These criteria were created for group A Streptococcus (*Streptococcus pyogenes*). Our patient fulfilled the Working Group’s criteria, except that the blood culture was positive for group B Streptococcus (*Streptococcus agalactiae*). Numerous studies demonstrate the importance of early detection and antibiotic treatment for GBS infections in general and early surgical management for necrotizing soft tissue infections (NSTIs) such as necrotizing fasciitis.

## INTRODUCTION

*Streptococcus agalactiae,* also called Group B *Streptococcus* (GBS) originates as a commensal organism of the intestinal microbiome before colonizing the urogenital tract. It is most commonly attributed to infection in neonates and pregnant women, but can also affect nonpregnant adults, particularly those with medical underlying conditions.^1^ In the 1970s, infants with early-onset disease (EOD) caused by GBS infection in the first week of the infant’s life suffered from case-fatality rates of up to 50%.^2-4^ In a systemic review and meta-analysis of close to 300,000 pregnant women across 390 studies, it was estimated that there is an 18% worldwide prevalence of maternal GBS colonization isolated from the vagina, rectum, or perianal area.^5^ Major complications include preterm delivery, hypoxic-ischemic encephalopathy, and newborn sepsis (both early- and late-onset).^5,6^

Guidelines recommend screening from 36 0/7 to 37 6/7 weeks, according to 2019 recommendations from the American College of Obstetricians and Gynecologists (ACOG). This is a change from 35-37 weeks stated in the CDC’s 2010 guidelines.^2^ Due to intrapartum prophylaxis recommendations such as these, developed nations have experienced a decrease in GBS infections.^7,8^ In 1990, the incidence of GBS disease was reported to be 1.8 per 1,000 live births.^2,9^ By 2006, the incidence of early-onset disease (EOD), which occurs during the first week of the infant’s life, was 0.37 per 1,000 live births and declined even further by 2015 to 0.33 per 1,000 live births.^2,10^

The incidence of invasive GBS disease in nonpregnant adults per 100,000 persons was reported to more than double from 3.6 in 1990 to 7.3 in 2007 and has continued to rise, with more recent data showing an increase from 8.1 in 2008 to 10.9 in 2016.^11-13^ Risk factors include advancing age, diabetes mellitus, malignancy, and immunosuppression.^7^ 2016 rates were 60% more than invasive group A streptococcal infections and 20% more than invasive infections caused by *Streptococcus pneumoniae*.^13-15^

For nonpregnant adults, there has been an increase in invasive GBS infections largely due to the increased prevalence of chronic medical conditions.^7,16^ The risk of developing invasive GBS disease is increased in patients with diabetes mellitus, neurological conditions, cirrhosis, stroke, cancer, and decubitus ulcers.^17^ Previous studies have demonstrated that the relative risk for GBS infections among nonpregnant adults is especially high for those with diabetes mellitus (RR = 30), HIV (RR = 30), and cancer (RR= 16.4).^7,18,19^ 5% of patients will also have a second relapsed infection on average 13 weeks after the first infection if caused by the same strain and 43 weeks for a different strain.^7,20^

Elderly adults have a far higher incidence, increasing 20% from 21.5 in 1999 to 26.0 in 2005.^21^ In the 1990-2007 period, the incidence for adults aged 65-79 years increased by 114.7%, larger than any other age group.^11,12^ From 2008-2016, the incidence increased from 19 to 24 for adults ages 65-79 years and from approximately 33 to 41 for adults ages 80 years or older.^21^

Nosocomial infections are a frequent cause of GBS disease.^17^ The use of medical instruments such as endoscopes and catheters during procedures is also a risk factor.^7^ A case-control study of 219 nonpregnant patients with invasive GBS and 645 hospital matched controls, found that 22% of the infected had a nosocomial GBS infection. The study showed that the placement of a central venous line corresponded to an odds ratio of 30.9 for those that were infected compared to the uninfected.^22^ This supports the understanding that iatrogenic causes play a significant role in infections originating from the skin or mucosal surfaces of patients.^7^

Since GBS isolates most commonly originate from the urethra, vagina, cervix, rectum, and perineal region, sexual contact is likely another major transmission route.^7^ In a study of 120 sexually intimate couples where at least one individual was colonized by GBS, 86% of the 57 couples that were both colonized had the same strain.^7,23^

Bacteremia and skin soft tissue infections are the most common invasive infections in nonpregnant adults.^11^ Other presentations include pneumonia, urosepsis, osteoarticular infection, meningitis, and endocarditis. The source of bacteremia may be from endocarditis, meningitis, skin and soft tissue infections, pneumonia, osteomyelitis, urinary tract infections, and infected intravascular catheters.^17^ Rarely, GBS infection has a fulminating pyrogenic exotoxin-mediated course characterized by acute onset multiorgan failure, shock, and sometimes death, referred to as toxic shock-like syndrome (TSLS).^11^

Toxic shock syndrome (TSS) was first described in 1978 by Todd et al.^24^ in a report of 7 pediatric patients where a prospective review of five patients isolated *Staphylococcus aureus* from either mucosal or sequestered areas rather than blood, which produces phage-group I rather than phage-group II.^24,25^ The most prominent features of the condition are acute onset hypotension, fever, and multiorgan failure.^26^

While TSS has been attributed to the use of tampons by menstruating women, its incidence has drastically decreased due to changes in the recommended use of tampons and tampon construction. The incidence of staphylococcal TSS is reported to be 0.5 cases/100,000 persons; however, in the 1980s, the incidence was 6.2-12.3 cases /100,000 persons before these changes.^24,27,28^ Currently, about half of the cases of TSS are nonmenstrual and instead are related to osteomyelitis, arthritis, wound infections, skin lesions, and barrier contraceptives.^29^

Staphylococcal TSS is most commonly attributed to the superantigen TSS toxin-1 (TSST-1).^29^ Other *S. aureus* protein exotoxin superantigens involved in TSS are staphylococcal enterotoxins (SEs) and staphylococcal enterotoxin-like toxins (SEls).^26,29^ TSST-1 is able to penetrate mucosal barriers and is involved in over 95% of cases of menstrual TSS. This is facilitated by staphylococcal cytolysin ⍺-toxin, which leads to IL-6, IL-1β, and TNF-⍺ release, causing mucosal disruption and facilitated TSST-1 penetration. TSST-1 is involved in approximately 50% of cases of nonmenstrual TSS, and staphylococcal enterotoxins SEB (most commonly), SEC, SEG, and SEI account for the other half of cases.^26^

Antibodies to TSST-1 are developed by adolescence in 70-80% of people and by adulthood in 90-95% of individuals.^29^ Those lacking antibodies to TSST-1 are particularly susceptible to developing complications such as TSS, as 90.5% of patients with menstrual TSS do not have antibodies to TSST-1 as measured by serum in the first week of the condition.^26,29,30^

The term “toxic shock-like syndrome” (TSLS) was reported in a 1987 report by Cone et al.^31^ to describe streptococcal toxic shock syndrome (STSS). It reported two patients infected by group A streptococcus (GAS) with symptoms similar to toxic shock syndrome caused by staphylococcal species.^31,32^ It was characterized in a 1989 series with 20 patients by Stevens et al.^33^ as having shock, multiorgan involvement, and necrotizing fasciitis caused by invasive group A streptococcal infections, 30% of which had a fatal outcome despite adequate treatment.^31,33^ In this series, Stevens et al.^33^ reported that of the 10 GAS isolates, 80% produced exotoxin A and that M proteins types 1 and 3 were the most common.^31,33^ The incidence of streptococcal toxic shock syndrome is reported to be 0.4 cases /100,000 persons.^24,27^

Streptococcal pyrogenic exotoxins (SPEs) A, B, C, F are erythrogenic, pyrogenic, and cytotoxic and are known to lead to the rash in scarlet fever. SPEs A, C, and F are superantigens that cause widespread, nonspecific T-cell proliferation, cytokine synthesis and release, and fever. M proteins bind to the host fibrinogen, inhibiting complement binding to peptidoglycan, which inhibits phagocytosis.^34^

Due to the medical community’s recognition that severe GAS infections and TSLS were increasing in incidence, the Working Group on Severe Streptococcal Infections sought to develop specific criteria for diagnosing streptococcal TSS. What was striking to investigators in The Working Group was that patients across various studies showed early onset shock, organ failure, and lack of apparent infection after a nonspecific prodromal illness, which was not commonly characterized by GAS infections. It recognized early shock and multiorgan failure as two factors that differentiated STSS from other invasive GAS infections.^31^

The Working Group’s specific case definition criteria were based on 1) the isolation of GAS and 2) clinical signs indicated by hypotension and evidence of organ involvement. The six possible signs of organ involvement were defined by specific criteria for renal impairment, coagulopathy, liver involvement, adult respiratory distress syndrome, macular rash appearing erythematous and widespread, and soft-tissue necrosis. The Working Group identified a definite case as one with 1) isolation of GAS from a normally sterile site and 2) both hypotension and two or more of the six signs for severe infection leading to shock and organ system involvement. They identified a probable case as 1) isolation of GAS from a nonsterile site and 2) both hypotension and two or more of the six signs for severe infection, assuming that there was not another identified etiology (Table 1).^31^

**Table 1:** Comparison of The Working Group of Severe Streptococcal Infection's "Proposed Case Definition for the Streptococcal Toxic Shock Syndrome" and the patient case. A definite case meets criteria IA and II (both IIA and IIB). A probable case meets criteria IB and II (A and B), assuming no other identified etiology^31^

Criteria	Working Group on Severe Streptococcal Infections' Proposed Case Definition	Patient Case
I	GAS isolation	
IA	GAS isolation from a normally sterile site	GBS isolation from a normally sterile site (blood)
IB	GAS isolation from a normally non-sterile site	
II	Indicators of severe infection (i.e., shock and organ system involvement)	
IIA	Hypotension: Systolic blood pressure ≤ 90 mmHg (adults) or < 5th percentile for age (children)	Systolic blood pressure between 40-70 mm Hg
	AND	
IIB	≥ 2 signs:	
IIB1	Renal impairment: creatinine ≥ 2mg/dL for adults or ≥ 2 times the upper limit of normal for age	Creatinine: 2.47 mg/dL
IIB2	Coagulopathy: platelets ≤ 100 x 10^9^/L or disseminated intravascular coagulation	Platelets: 25 x 10^9^/L, Prothrombin time (PT): 82.9 sec (Reference: 12.5-14.5 sec), International normalized ratio (INR): 9.67 (Reference: 1.12-1.46) Partial thromboplastin time (PTT): 44.5 sec (Reference: 25.0-35.0 sec)
IIB3	Liver involvement: ALT, AST, or total bilirubin ≥ 2 times the upper limit of normal for age or ≥ 2 times baseline for preexisting liver disease	Alanine aminotransferase (ALT): 1,471 U/L Aspartate aminotransferase (AST): 7,359 U/L
IIB4	Acute respiratory distress syndrome, diffuse capillary leak, or pleural/peritoneal effusions with hypoalbuminemia	
IIB5	Generalized erythematous macular rash (may desquamate)	Widespread erythematous rash
IIB6	Soft-tissue necrosis (e.g., necrotizing fasciitis, myositis, gangrene)	Necrotizing fasciitis

## CASE REPORT

**Figure 1:**
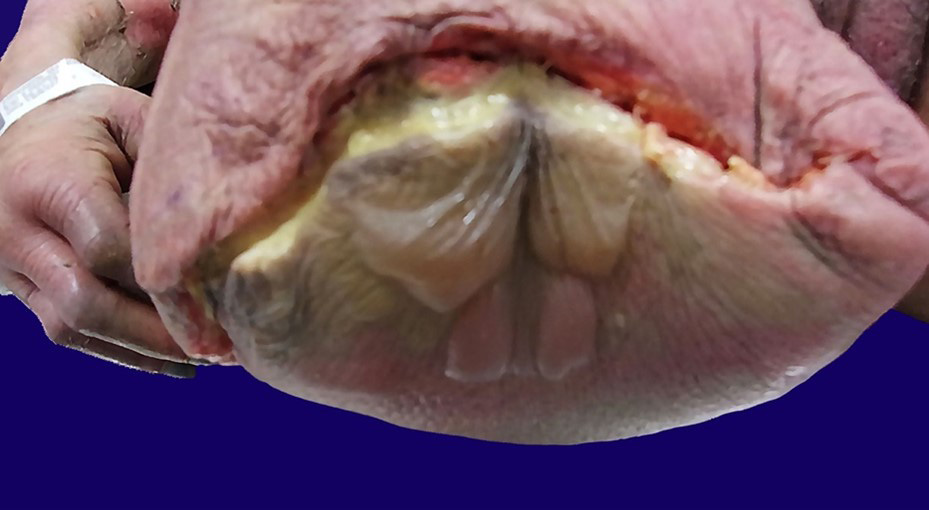
Gross image of left surgical stump from below-knee amputation showing bullae characteristic of Type II NF and dusky discoloration.

A 77-year-old hypertensive female with uncontrolled type 2 diabetes mellitus and a history of bilateral foot ulcers presented to the hospital in septic shock. Prior to admission, systolic blood pressure ranged from 40-70 mmHg and improved to 90-100 mmHg after being administered intravenous fluids. At admission, she was hypotensive with WBC 28.59x10^3^ cells/mm^3^; necrotic tissue around the foot ulcers was evident. At the time of physical examination, her blood pressure was 106/56 mmHg, pulse 90/min, respiratory rate 15/min, temperature 35.9°C, and oxygen saturation 98% on 2L nasal cannula. The patient was frail-appearing but not in acute distress and was oriented to person, place, and time. A clinical diagnosis of necrotizing fasciitis was made and she underwent bilateral lower limb amputations (Figure 1). The patient met criteria for SIRS, septic shock, and multi-organ dysfunction (Table 2).

**Table 2:** SIRS, Sepsis, and Septic Shock Criteria compared to patient findings^35^

**Criteria**	**Patient**
**SIRS (≥2 meets criteria)**	
Temp >38°C or <36°C	Temp 35.9°C (Day 0)
HR >90/min	HR up to 178/min (Day 0)
WBC >12,000/mm^3^, <4,000/mm^3^, or >10% bands	WBC 28.59x103 cells/mm^3^ (Day 0)
**Sepsis (SIRS + Source of Infection)**	
Suspected/present infection source	Two blood cultures positive for GBS (Day 0)
**Severe Sepsis (Organ Dysfunction, Hypotension, or Hypoperfusion)**	
Lactic acidosis, SBP <90 or SBP drop ≥40 mm Hg of normal	SBP 40-70 mmHg (Day 1)
**Septic Shock**	
Severe sepsis with hypotension, despite fluid resuscitation	SBP 85 mmHg (Day 2)
**Multiple Organ Dysfunction Syndrome**	
Evidence of ≥2 organs failing	Cr 2.47 mg/dL and AST 7359 U/L (Day 3)

**Figure 2:**
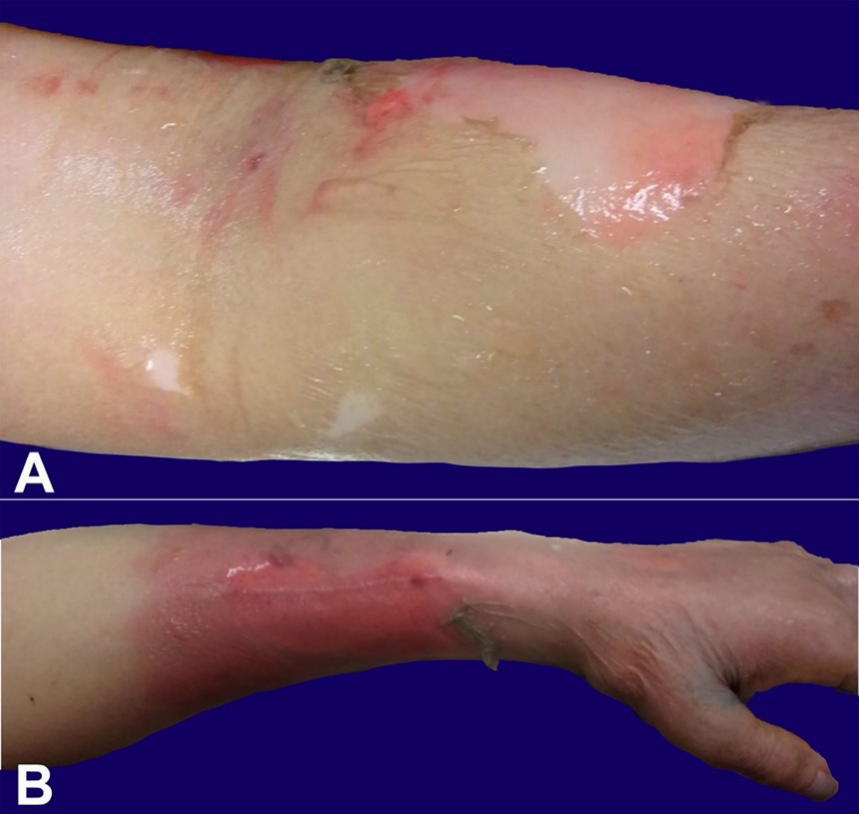
**A–** Gross pathologic examination of right upper limb erythema and desquamation; **B –** Gross pathologic examination of left upper limb erythema and desquamation.

**Figure 3:**
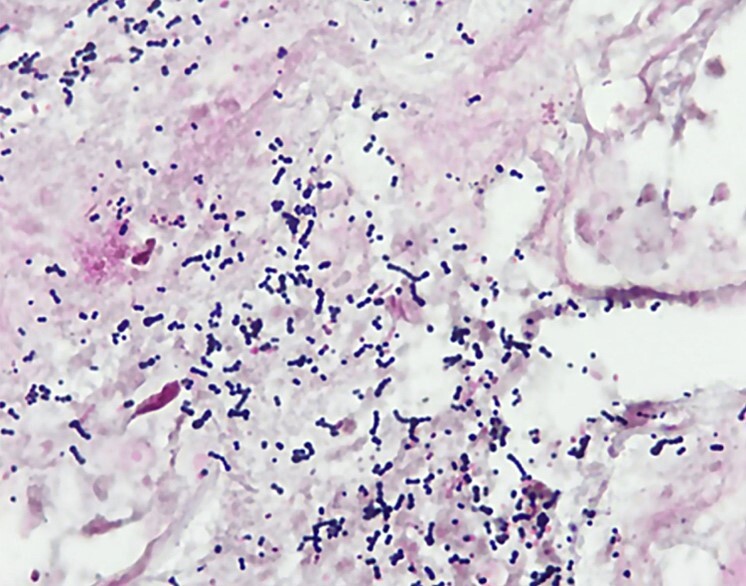
Microphotography showing sheets of Gram-positive cocci in chains (Gram stain, 100X).

Grossly, soft tissue exhibited the classic dermatologic clinical manifestation of sepsis and septic shock characterized by eroded bullous lesion with an erythematous base (Figures 2A and 2B). Microscopically, fascia was necrotic without neutrophils, and Gram stain revealed sheets of Gram-positive cocci (Figure 3).

**Figure 4:**
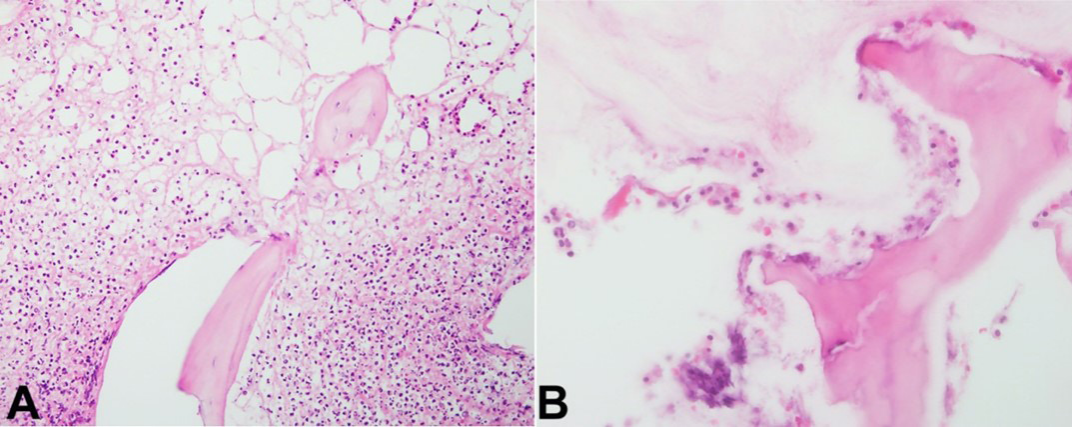
**A –** Microscopic image of amputation specimen showing osteonecrosis with empty osteocyte cavities surrounded by sheets of neutrophils indicative of acute osteomyelitis (H&E stain 100X); **B –** High power image of osteonecrosis with empty osteocyte cavities, irregular edges, and adjacent neutrophils some of which appear apoptotic (H&E stain, 400X).

These findings reflected histopathologic stage III necrotizing fasciitis, which is associated with 47% mortality.^36,37^ Acute osteomyelitis was evident on microscopic examination following amputation (Figures 4A and 4B).

Blood culture was positive for *Streptococcus agalactiae* (GBS)*.* Autopsy showed a similar histology of stage III necrotizing fasciitis involving the surgical stump. Bilateral erythema and desquamation of the upper limbs (Figures 2 and 3) and multiorgan failure, notably renal impairment, coagulopathy, and liver impairment, met the clinical picture and fulfilled the criteria for STSS due to Group A Streptococcus (GAS) defined by The Working Group on Severe Streptococcal Infections (Table 1).^31^ GBS rather than GAS was isolated from the patient’s blood. Other than this, all the criteria were met since Criteria IA, IIA, and >2 signs indicating severe organ system involvement (IIB1, IIB2, IIB3, IIB5, and IIB6) were confirmed in our patient with necrotizing fasciitis (Table 3).

**Table 3:** Selected patient labs during hospital course

	RR	Day 0	Day 1	Day 2	Day 3
Partial thromboplastin time (R)	25.0-35.0	32.6	41.7	44.9	44.5
Prothrombin time INR	1.12-1.46	1.42	2.58	3.2	9.67
Platelets (10^9^/L)	177-406	245	263	132	25
Hemoglobin (g/dL)	14.6-17.8	6.8	9.4	9.0	5.4
Leukocytes (10^3^/µL)	3.2-10.6	23.17	28.52	22.01	46.21
Creatinine (mg/dL)	0.6-1.2	0.69	0.63	1.37	2.47
Aspartate aminotransferase (U/L)	0-50	8		7467	7359
Alanine aminotransferase (U/L)	0-55	8		1217	1471

RR= reference range.

## DISCUSSION

While TSLS (also referred to as streptococcal TSS) generally describes conditions caused by *Streptococcus pyogenes* (GAS), our patient fulfilled The Working Group’s criteria with an infection caused by *Streptococcus agalactiae* (GBS). The patient fulfilled the criteria I as GBS (rather than GAS) was isolated from a normally sterile location. Criteria IIA was also fulfilled as systolic blood pressure was in the range between 40-70 mm Hg while being transported to the hospital.

Criteria IIB was fulfilled as five of the six signs associated with severe infection were present: 1) Renal impairment (IIB1) was indicated by creatinine levels of 2.47 mg/dL; 2) Coagulopathy (IIB2) was indicated with a platelet level of 25x10^9^/L, prothrombin time (PT) of 82.9 sec, international normalized ration (INR) of 9.67, and partial thromboplastin time (PTT) of 44.5 sec; 3) Liver involvement (IIB2) was indicated by alanine aminotransferase (ALT) levels of 1,471 U/L and aspartate aminotransferase (AST) levels of 7,359 U/L; 4) A widespread erythematous rash (IIB5) was present; and 5) Soft tissue necrosis (IIB6) was fulfilled by the diagnosis of necrotizing fasciitis. This met the Working Group’s criteria for a definite case as it fulfilled criteria IA and II (including IIA and IIB).

GBS infections are an increasing problem in older adults and those with chronic medical conditions, especially diabetes mellitus.^7^ Surgical management of chronic ischemic medical conditions in an aging United States population places an increasing number of patients at risk for invasive GBS infections.^17^

Over 40% of invasive GBS infections and over 50% of deaths from GBS infections occur in elderly adults 65 years of age or older.^38^ As the skin and mucosa serve as the first line of defense against GBS, chronic conditions such as diabetes, pressure ulcers, and peripheral artery disease, associated with cutaneous and mucosal infections, allow GBS to migrate from its reservoir.^7^

In elderly adults, lower levels of type-specific antibodies to the capsular polysaccharide (CAP) may lead to susceptibility to invasive GBS infections.^7^ A study of 40 healthy adults 65 years or older showed that 36 had sera without neutrophil-mediated functionality. When administered sera from young adults vaccinated with a type V conjugate vaccine, all demonstrated neutrophil-mediated functionality against type V GBS.^7,39^ Type V GBS conjugate vaccines given to elderly individuals have also been shown to increase type V immunoglobulins 4, 8, 26, and 52 weeks after vaccine immunization in a study comparing 22 elderly adults given a type V-tetanus toxoid conjugate vaccine compared with 10 elderly adults given a tetanus-diphtheria toxoid vaccine.^7,40^ Administration of type II-specific antibodies also showed improvement in opsonophagocytosis in individuals with insulin-dependent diabetes.^7,41^

Patients with diabetes account for 20-25% of GBS disease in nonpregnant adults.^17^ A hyperglycemic state is often attributed to increased susceptibility to bacterial infections in these patients. Hyperglycemic states have been shown to reduce both neutrophils’ opsonophagocytosis (OP) activity and superoxide dismutase (SOD) production.^7,42^ In a study of 10 adults with type 2 diabetes mellitus, it was found that for baseline glycemic conditions, their neutrophils did not have defective OP activity. When placed in either 60 mM glucose (upper range during diabetic ketoacidosis), 120 mM glucose (representing modest hyperglycemic levels), or 60mM choline chloride (a GBS osmolyte), it was shown that both OP and SOD production decreased under hyperglycemic conditions and that administration of type III antibodies improved OP.^42^

Necrotizing fasciitis caused by *S. agalactiae* has been rarely described in the literature, can mimic Streptococcal Toxic Shock, and may result in a fatal outcome.^17,43,44^ A 2016 case report on a male with diabetes and bilateral GBS necrotizing fasciitis of the foot made the argument that since GBS was isolated both from blood and bilateral extremities, hematogenous spread can allow GBS to cause secondary necrotizing fasciitis in remote locations. The authors noted a 1-week delay in the secondary necrotizing fasciitis, which it attributed to the rate of inflammation developed in the remote fascia and subcutaneous tissue. It also identified 22 cases of GBS necrotizing fasciitis, including the case it was reporting. Of these 22 cases, 73% had diabetes mellitus, and 14% had cancer. 70% of the patients had necrotizing fasciitis in the lower extremity, and there was a 19% mortality rate among those with a known prognosis.^45^

The cell wall of *Streptococcus agalactiae* contains two types of carbohydrate antigens: the group B antigen, and the capsular polysaccharide antigen.^1^ The group B antigen is found in all strains of *Streptococcus agalactiae*, which is why it is also called Group B Streptococcus. There are 10 serotypes of GBS, Ia, Ib, and II-IX, which are characterized based on surface capsular polysaccharide antigens and have a distribution that varies over geographic regions and with time.^46^ Surface proteins such as C-protein isolates are also used for strain characterization. Serotypes Ia, III, and V account for approximately 70% of invasive infections in nonpregnant adults.^7^

For pregnant women worldwide, 98% of GBS isolates are of serotypes I-V. Serotype III has the greatest association with invasive GBS disease and the hypervirulent clonal complex (CC) 17, which may lead to late-onset GBS disease and meningitis. Serotype III is noted to be less prevalent in Asia and may account for the lower GBS disease incidence among pregnant women in Asia (12.5% in Asia vs. 18% worldwide). Further development of our understanding of colonization prevalence and serotype distribution could lead to a better understanding of how invasive GBS infections in newborns vary across regions of the world.^5^

Levels of type-specific antibodies to the capsular polysaccharide (CAP) have been linked with the risk of systemic GBS infection. In a study comparing the sera of 111 infants that had acute type III GBS infection with 45 healthy infants of mothers with type III GBS colonization at delivery, it was found that those with acute infections had a concentration of antibodies to type III GBS of less than 1.7 µg/mL and a median of 0.4 µg/mL, which was significantly lower than the healthy infants which had concentrations over 2 µg/mL and a median of 12.6 µg/mL.^47^

GBS virulence factors include: (a) the exopolysaccharide capsule, which inhibits complement deposition and activation, thereby reducing opsonophagocytic clearance, and (b) surface-associated toxin (β hemolysin/cytolysin), which is associated with direct lysis of cells.^7^ GBS produces an uncharacterized pyrogenic toxin(s), explaining the ability of GBS to cause TSLS.^11^

Different levels of expression of the exopolysaccharide capsule may correlate with different functions. Low levels lead to tissue injury and inflammation, whereas high levels lead to phagocytic resistance.^48,49^ Sialic acid, a component of the capsule, prevents C3b deposition to inhibit opsonization.^7,50^

β-hemolysin/cytolysin (β-h/c) causes the formation of pores, which enables cell lysis. This may manifest as soft tissue damage or damaged cardiomyocytes, endothelial cells, and lung epithelial cells. It has been correlated with the severity of septic arthritis, as 3% of cases of septic arthritis contain isolates of GBS.^48,51^ It has also been suggested that β-h/c may cause apoptosis of macrophages and monocytes.^7,52,53^

The C-protein complex includes surface proteins Cα (*bca* gene), Rib (*rib* gene), and Cβ (bac gene) and promotes adhesion to host cells.^1,54,55^ β-antigen, a protein C-component, enables binding of the Fc fragment on IgA, which inhibits it from interacting with Fc receptors (on neutrophils, macrophages, and eosinophils) and therefore enables protection from host immune clearance.^7,56,57^

Adhesins such as protein FbsA, a fibrinogen-binding protein, allows GBS to adhere to the cerebral endothelium, contributing to the development of meningitis.^1,58,59^ C5a, a serine protease encoded by the *scp*B gene, inactivates the chemotactic C5a, leading to decreased neutrophil recruitment and inflammation.^1,60^ Other factors of GBS include but are not limited to lipoteichoic acid (LTA), superoxide dismutase (SOD), penicillin-binding protein 1a, hyaluronate lyase, CAMP factor, and metalloproteinases.^7^ Aside from virulence factors, other factors that enable avoidance of host immunity include antigenic variation of C-protein complex components, capsular serotype switching, and macrophages survival.^7,61-63^

Early recognition of infection and treatment with appropriate antibiotics and, in some cases, aggressive surgical intervention are essential for successful management.^17^ In a systematic review and meta-analysis of 109 studies that included 6,051 patients with necrotizing soft tissue infections (NSTIs),, analysis showed an advantage of surgical treatment within 6 hours of patient presentation compared to over 6 hours in reducing the mortality rate with an odds ratio of 0.41.^64^ A systemic review of 287 articles with 341 patients with NSTIs that received surgical debridement was conducted to further investigate the optimal timing for surgical management noting that literature recommendations ranged widely from within 3 hours to 36 hours of diagnosis. It found a mortality rate of 14% for the 143 patients with initial debridement <12 hours after diagnosis compared to a mortality rate of 25.8% for the 198 patients with initial debridement ≥12 hours after diagnosis.^65^

## CONCLUSION

Group B Streptococcal Toxic Shock-Like Syndrome may have a similar outcome to STSS caused by GAS and other pathogens, and in limited studies, mortality has been reported as 30% or greater.^31^ Treating TSLS caused by invasive GBS disease may also prove to be a challenge, as it has been reported that the minimum inhibitory concentration of penicillin for *S. agalactiae* is 4-8 times higher than that of *S. pyogenes*.^17^ However, studies showing the benefits of type-specific antibody therapy and GBS vaccines to prevent neonatal illness provide hope for developing and approving vaccines to prevent invasive GBS disease for neonates, pregnant women, and high-risk nonpregnant adults.^7,17,39,40,41,66,67^
